# Antiviral activity of gliotoxin, gentian violet and brilliant green against Nipah and Hendra virus *in vitro*

**DOI:** 10.1186/1743-422X-6-187

**Published:** 2009-11-04

**Authors:** Mohamad Aljofan, Michael L Sganga, Michael K Lo, Christina L Rootes, Matteo Porotto, Adam G Meyer, Simon Saubern, Anne Moscona, Bruce A Mungall

**Affiliations:** 1 Australian Animal Health Laboratory, CSIRO Livestock Industries, Geelong, Australia; 2 Departments of Pediatrics and of Microbiology and Immunology, Weill Medical College of Cornell University, New York, NY, USA; 3 Measles, Mumps, Rubella and Herpes virus Laboratory Branch, Division of Viral Diseases, Centers for Disease Control and Prevention, Atlanta, Georgia, USA; 4 School of Veterinary Science, The University of Queensland, St Lucia, Australia; 5 CSIRO Molecular and Health Technologies, Clayton, Australia

## Abstract

**Background:**

Using a recently described monolayer assay amenable to high throughput screening format for the identification of potential Nipah virus and Hendra virus antivirals, we have partially screened a low molecular weight compound library (>8,000 compounds) directly against live virus infection and identified twenty eight promising lead molecules. Initial single blind screens were conducted with 10 μM compound in triplicate with a minimum efficacy of 90% required for lead selection. Lead compounds were then further characterised to determine the median efficacy (IC_50_), cytotoxicity (CC_50_) and the *in vitro *therapeutic index in live virus and pseudotype assay formats.

**Results:**

While a number of leads were identified, the current work describes three commercially available compounds: brilliant green, gentian violet and gliotoxin, identified as having potent antiviral activity against Nipah and Hendra virus. Similar efficacy was observed against pseudotyped Nipah and Hendra virus, vesicular stomatitis virus and human parainfluenza virus type 3 while only gliotoxin inhibited an influenza A virus suggesting a non-specific, broad spectrum activity for this compound.

**Conclusion:**

All three of these compounds have been used previously for various aspects of anti-bacterial and anti-fungal therapy and the current results suggest that while unsuitable for internal administration, they may be amenable to topical antiviral applications, or as disinfectants and provide excellent positive controls for future studies.

## Background

Nipah (NiV) and Hendra (HeV) viruses are two newly emerging zoonotic paramyxoviruses that are lethal to humans. HeV was first isolated during two outbreaks of respiratory illness in horses in Australia [1]. Highly lethal in horses, these initial HeV outbreaks also resulted in two human fatalities, including one person initially presenting with a flu-like illness followed by apparent recovery who subsequently died one year later due to meningoencephalitis [2]. HeV has continued to re-emerge in eastern Australia with more than twelve separate outbreaks being documented [3] resulting in over 30 equine deaths and an additional human fatality in each of August 2008 [4] and August 2009 [5]. The initial NiV outbreak occurred in peninsular Malaysia in 1998 and by June 1999, more than 265 cases of encephalitis, including 105 deaths, had been reported in Malaysia and 11 cases of disease with one death in Singapore [6]. In addition to the human health impact, the economic impact of this disease was dramatic. Containment procedures resulted in the slaughter of almost 1.2 million pigs and the virtual closure of the pig farming industry in Malaysia. Electron microscopy, serologic, and genetic studies indicated that this virus was a paramyxovirus, subsequently named NiV after the village in Malaysia from which one of the first isolates was obtained from the cerebrospinal fluid of a fatal human case [6,7]. Serological surveillance and virus isolation studies indicated that NiV resides naturally in flying foxes in the genus Pteropus (reviewed in [8]). NiV has continued to re-emerge in Bangladesh causing fatal encephalitis in humans and for the first time, person-to-person transmission appeared to have been a primary mode of spread [9-14]. In addition, there appeared to be direct transmission of the virus from it's natural host, the flying fox, to humans, and the case mortality rate was ~70%; significantly higher than any other NiV outbreak to date.

A number of recent reports of potential vaccine approaches [15-19] and experimental therapeutics [19-25] have been described, however, there is still no vaccine or antiviral treatment specifically indicated for either HeV or NiV infections (reviewed in [26]). An open-label trial of ribavirin in 140 patients during the initial NiV outbreak in Malaysia showed ribavirin therapy was able to reduce mortality of acute NiV encephalitis [27]. While this study reported no serious side effects, ribavirin has been associated with a range of side effects primarily related to haemolytic anaemia [28]. The antiviral efficacy of ribavirin has also been demonstrated against HeV and NiV *in vitro *[29,30]. *In vivo*, a recent study showed that the interferon inducer poly(I)-poly(C_12_U), but not ribavirin, was able to prevent mortality in five of six animals in a hamster model of NiV infection [31]. Recently, we described the antiviral properties of chloroquine against Henipaviruses *in vitro *[32], although a recent study reported no anti Henipavirus effects in a ferret model *in vivo *[33].

There have also been a number of recent reports describing the development of surrogate assays to screen and evaluate HeV and NiV antivirals or perform serological surveys at biosafety level 2 (BSL2) [24,25,34-37]. These pseudotyped assays provide excellent surrogate BSL2 assays for the evaluation of virus entry and fusion mechanisms, enabling wider access for potential antiviral evaluation. Significantly, our recent description of chloroquine as an effective henipavirus antiviral was identified using a modified, multicycle pseudotype screening assay with efficacy subsequently confirmed against live virus [32]. This study demonstrates that surrogate assays can provide legitimate antiviral leads, however, these will ultimately require live virus confirmation. Mini-genome assays [23,38] may provide an effective complimentary approach to pseudotyped assays but ultimately, inhibitors identified using these approaches must also be validated against live virus at biosafety level 4 (BSL4). In an effort to expedite the process of antiviral development, we have recently described an immunoassay format amenable to high throughput screening (HTS) of antiviral compounds, directly against live HeV and NiV [30]. Using this live virus HTS approach, we have identified a number of potential antiviral compounds [39], three of which are commercially available, public access molecules. While these compounds may only have limited potential therapeutic uses, they provide an excellent group of positive controls with which to evaluate and standardise subsequent screening assays. To this end, in an effort to further validate surrogate assays for antiviral screening approaches, we have compared the efficacy of these compounds using our recently described multicycle replication pseudotype assay [32].

## Results

**Figure 1:**
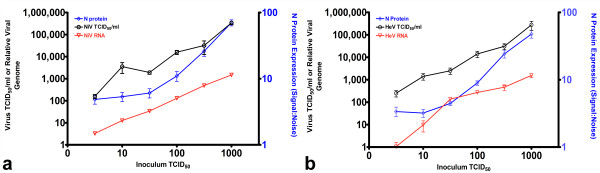
**Detection of NiV (a) and HeV (b) via end-point titration, Taqman PCR and immunodetection**. 1/2 log dilutions of virus (100 μl) were incubated with 20,000 Vero cells per well for 24 h at 37°C and 5% CO_2_. Virus released into the media was quantified by end-point titration and TCID_50_/ml (n = 3) determined using the Reed-Meunch method [73]. Monolayers were either fixed with methanol, air dried and immunostained with anti-NiV-nucleoprotein polyclonal antisera as previously described [30] or viral RNA was extracted from cells (n = 3) and Taqman PCR was used to quantitative the relative expression of the N gene [17,23]. S/N, signal:noise ratios calculated as signal/background values (n = 15). Values are expressed as the mean +/- S.E.

**Figure 2:**
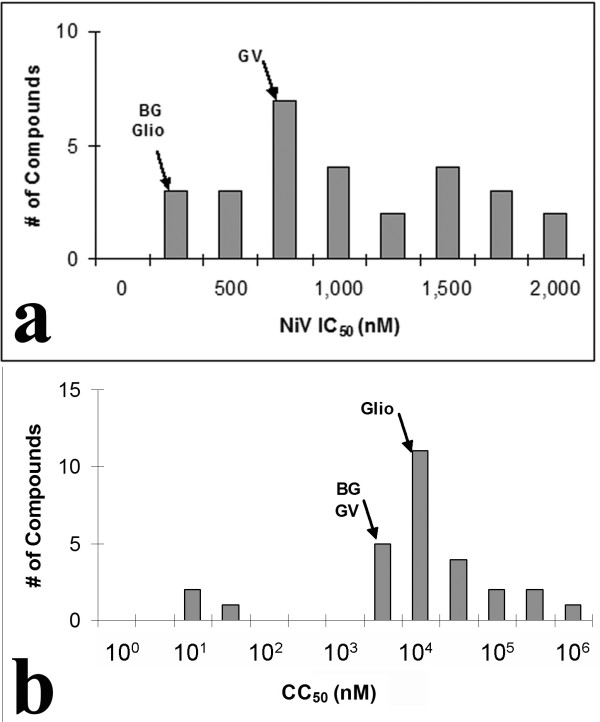
**HTS screening of a small compound library against live NiV**. Cells were treated with 10 μM of each compound (100 μl) immediately prior to infection with 1,000 TCID_50 _NiV in 100 μl. Cells were incubated overnight at 37°C. Monolayers were fixed with methanol, air dried and immunostained with anti-NiV-N polyclonal antisera as described above. **(a) **Dose response curves were generated for 28 compounds with >90% inhibition (10 μM) showing a range of 50% inhibitory concentration (IC_50_) values all less than 2 μM (n = 3). **(b) **A wide range of 50% cytotoxicity concentration (CC_50_) values was observed for these 28 compounds (n = 3). Values determined for brilliant green (BG), gentian violet (GV) and gliotoxin (glio) are indicated.

**Figure 3:**
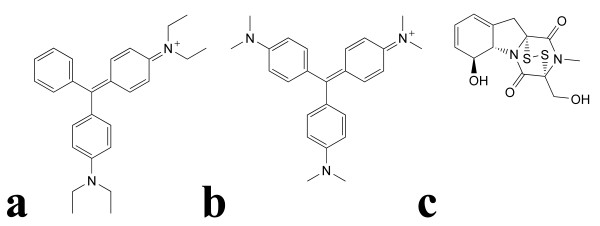
**Chemical structures of a. brilliant green, b. gentian violet and c. gliotoxin**.

Utilising a simple monolayer based assay amenable to HTS of antivirals directly against live virus [30], we performed a preliminary single blind screen of a library of 8,040 low molecular weight molecules. This assay incorporates immunological detection of the viral nucleoprotein (N) following infection and fixation of cell monolayers. We have previously demonstrated a linear relationship between N protein expression and viral inoculum [30], and for clarity, we have also directly compared the titer of infectious virus recovered from Vero cells with the level of N protein expression detected using this immunoassay approach (Figure 1). While the immunoassay is largely insensitive to changes in viral inoculum below 100 TCID_50_, there is a linear relationship between viral inoculum and protein expression for both HeV and NiV above 100 TCID_50 _comparable to that observed for viral RNA and infectious virus titers recovered from the same wells (Figure 1). Our initial screen was conducted using 1,000 TCID_50 _of each virus ensuring N protein expression was well within the linear portion of this curve and would be proportional to the levels of infectious virus recovered. This initial screen resulted in a predictable distribution of inhibition values with the majority of compounds exhibiting between 25 and 75% inhibition of NiV infection [38]. The primary screen of DMSO stocks revealed 54 compounds inhibiting NiV infection by greater than 90%. To confirm inhibitory activity 49 compounds were sourced from lyophilised stocks and redissolved in DMSO to be retested as fresh stocks. On retest, 28 of the compounds exhibited greater than 90% inhibition of NiV *in vitro*. Dose-response experiments were performed on each of these 28 compounds to determine the IC_50 _concentrations for each in addition to the CC_50 _determined in Vero cells (Figure 2). Upon unblinding, three of these remaining 28 lead compounds were identified as brilliant green, gentian violet and gliotoxin (Figure 3), commercially available compounds with a variety of historical applications. All three compounds were at the lower end of the range of IC_50 _values determined, but were also at the lower end of the CC_50 _range, indicating higher toxicity than many of the novel compounds identified (Figure 2).

All three compounds were shown to effectively inhibit both NiV and HeV (Table 1) infection. NiV IC_50 _values for brilliant green and gliotoxin were ten fold lower than ribavirin while gentian violet was four fold lower than ribavirin (Table 1). HeV IC_50 _values for brilliant green and gliotoxin were three fold lower than ribavirin while gentian violet was slightly less effective than ribavirin (Table 1). Incubation of compounds in parallel with virus inhibition assays reveals all three compounds are cytotoxic at high concentrations using both ATP based and resorufin based measures of cytotoxicity (Table 1). The concentration of compound exhibiting 50% cytotoxicity (CC_50_) for all three compounds was similar in Vero cells but varied more than three-fold in 293T cells reflecting the lack of correlation often observed between measures of cytotoxicity. Of note, all three compounds were considerably more cytotoxic than ribavirin in Vero cells. The therapeutic index (TI) for each compound indicates all three compounds are more amenable to inhibition of NiV than HeV (Table 1) but all have very narrow margins of safety. Confirmation of henipavirus inhibition was achieved with a recently described NiV-G-VSV-pseudotype assay which mimics multicycle replication [32] and the related HeV-G-VSV assay (Table 2). Additionally, antiviral efficacy was evaluated against the parent pseudotyped virus (VSV), HPIV3 and an influenza H1N1 virus (Table 2). The similar levels of inhibition observed for most of these viruses would indicate the antiviral activity of these compounds occurs by a process not specific to henipavirus entry. Of note however, only gliotoxin exhibited a dose-dependant inhibition of influenza virus (Table 2) suggesting brilliant green and gentian violet efficacy is not simply a product of viral envelope disruption. Both brilliant green and gliotoxin exhibited similar IC_50_s for each of the pseudotyped viruses, suggesting their action may be related to the VSV backbone, rather than the specific glycoproteins for each virus. Curiously, gentian violet displayed a striking selectivity for pseudotyped HeV inhibition, and to a lesser extent, pseudotyped NiV (Table 2). This unexpected result may potentially signal a more specific antiviral action attributable to gentian violet, or alternatively, an enhanced sensitivity of pseudotyped assay formats when compared to live virus assays. This may have considerable implications for the use of surrogate assay screens as the primary tools for antiviral discovery. A more detailed follow-up of this observation is currently underway.

**Table 1:** IC_50_, CC_50 _and therapeutic index (TI) values calculated for each compound against live Nipah and Hendra viruses.

	**Brilliant Green**	**Gentian Violet**	**Gliotoxin**	**Ribavirin**
	
NiV IC_50 _(nM)	218	525	149	3,897
	
HeV IC_50_(nM)	778	2,679	579	2,241
	
CellTiter-Glo^® ^CC_50_(nM)	4,672	5,865	4,896	149,745
	
alamarBlue^® ^CC_50_(nM)	861	2,828	1,609	ND^a^
	
NiV TI (CellTiter-Glo^®^)	21.39	11.16	32.81	38.42
	
NiV TI (alamarBlue^®^)	3.95	5.39	10.80	ND^a^
	
HeV TI (CellTiter-Glo^®^)	6.00	2.19	8.44	66.82
	
HeV TI (alamarBlue^®^)	1.11	1.06	2.78	ND^a^

^a ^ND = not determined, values are averages of at least 3 independent experiments.

**Table 2:** IC_50 _values calculated for pseudotyped Nipah (pNiV), Hendra (pHeV), VSV (pVSV), HPIV3 and Influenza viruses.

	**Brilliant Green**	**Gentian Violet**	**Gliotoxin**
	
pNiV IC_50 _(nM)	42	61	100
	
pHeV IC_50 _(nM)	34	0.3	366
	
pVSV IC_50 _(nM)	15	268	232
	
HPIV3 IC_50_(nM)	248	860	527
	
Influenza IC_50_(nM)	DNC^a^	DNC^a^	13,786

^a ^DNC = non-linear regression curve did not converge, values are averages of at least 3 independent experiments.

**Figure 4:**
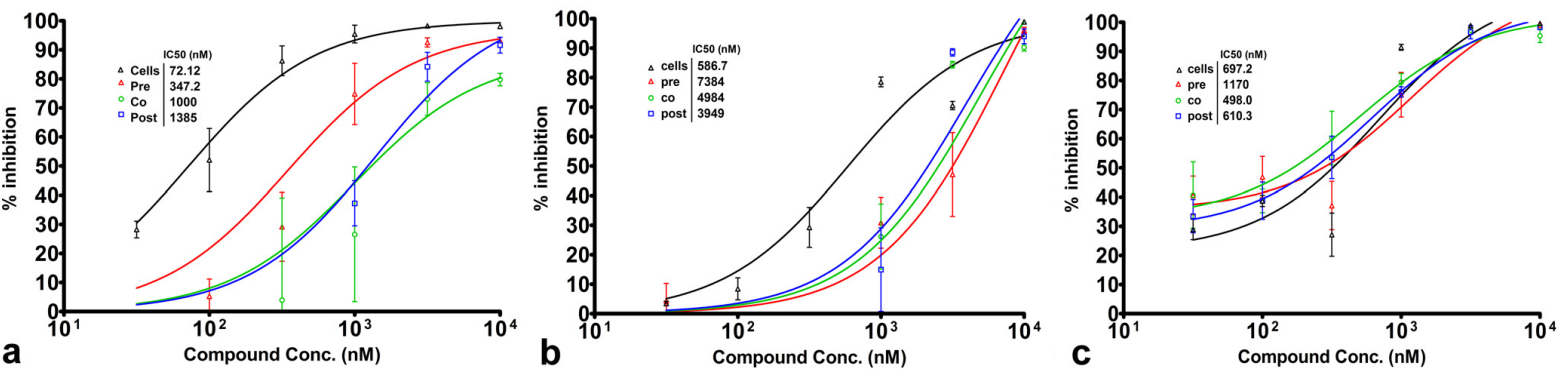
**Effect of time of addition of compounds on NiV antiviral activity for brilliant green (a) gentian violet (b) and gliotoxin (c)**. Inhibition of NiV infection as measured by viral nucleoprotein expression determined by immunoassay. For cell pre-incubation (Cells), each compound was incubated with cells for 60 min, media was then removed and virus inoculum (1,000 TCID_50_/ml NiV) added and incubated for 60 min, then inoculum was removed and replaced with EMEM-10 and cells were incubated overnight. For Pre-infection (Pre), virus was incubated with compound for 60 min, then added to cells for 60 min prior to replacement with EMEM-10 overnight. Co-infection (Co) wells received compound and virus inoculum simultaneously, incubated for 60 min, then media was replaced with EMEM-10 and cells were incubated overnight. Post-infection (Post) wells were infected with virus for 60 min followed by inoculum removal and replacement with compound in media. Cells were incubated and assayed as above. Values are expressed as the Mean +/- S.E (n = 3).

**Figure 5:**
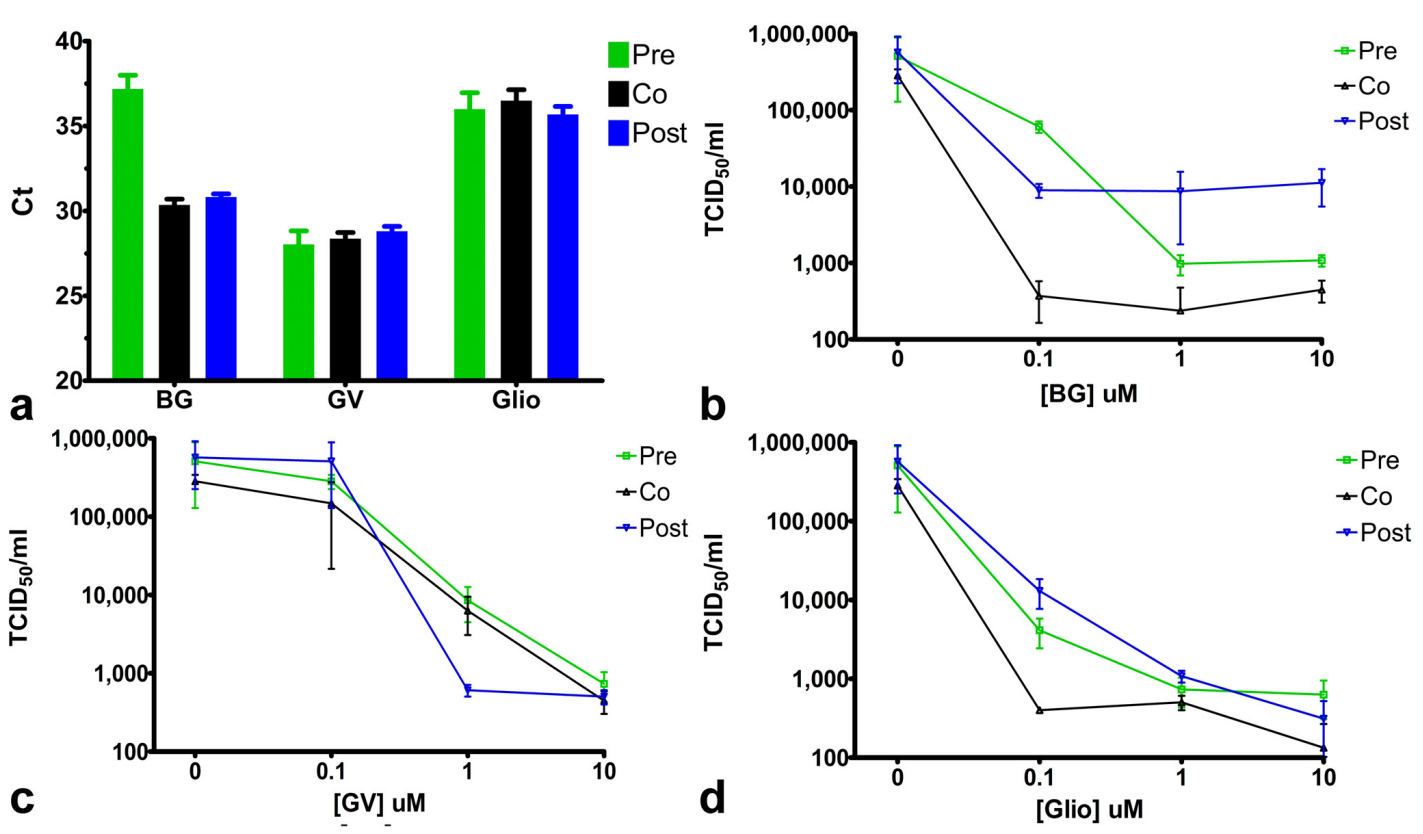
**Effect of time of addition of compounds on NiV genome replication and infectious titer**. **(a)** Relative N gene detection (Ct vs. treatment) determined by Taqman PCR for brilliant green (BG), gentian violet (GV) and gliotoxin (glio). Infectious NiV titers determined by end point titration in the supernatants of Vero cells treated with brilliant green **(b) **gentian violet **(c) **and gliotoxin **(d) **before, during and after virus infection overnight. Values are expressed as the Mean +/- S.E (n = 3).

Time of addition experiments indicated that preincubation of cells with either brilliant green or gentian violet prior to NiV infection resulted in more effective inhibition of viral protein expression than when compounds were added during or after virus infection (Figures 4a and 4b). This may be due in part to increased cytotoxicity associated with longer times of compound exposure to the cell monolayer, however, gliotoxin which exhibits similar levels of cytotoxicity, did not induce enhanced antiviral activity under the same conditions (Figure 4c). Preincubation of brilliant green with virus prior to viral infection also resulted in enhanced inhibition of viral protein expression (Figure 4a), viral genome expression (Figure 5a) and release of infectious virus (Figure 5b) suggesting a direct effect on viral particles. Gliotoxin and gentian violet efficacy appeared independent of the time of addition suggesting they may be exerting their effects subsequent to virus binding and entry. Similar results were observed with time of addition experiments during HeV infection but are not shown for brevity.

**Figure 6:**
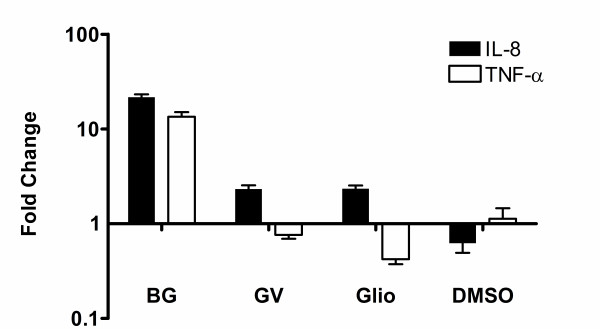
**Expression of IL-8 and TNF-α following treatment with brilliant green, gentian violet or gliotoxin**. Vero cell monolayers in 48 well plates were treated with 1 μM compound overnight followed by RNA extraction, DNAse treatment, reverse transcription and real time PCR assay using SYBR green. Gene expression was normalised using GAPDH expression and is expressed as a fold change relative to untreated wells (Mean ± S.E.M.).

As an indication of the effect of these compounds on the cellular inflammatory response, an evaluation of the induction of the cytokines IL-8 and TNF-α was also performed. Real Time PCR revealed brilliant green (1 μM) strongly induced both IL-8 and TNF-α expression fifteen to twenty fold (Figure 6). In contrast, gliotoxin suppressed TNF-α expression with mild (two fold) induction of IL-8 by both gentian violet and gliotoxin compared to DMSO treated control cells (Figure 6).

## Discussion

We have recently described a reliable and sensitive HTS method that potentially allows the screening of large libraries of compounds for antiviral drug discovery *in vitro *[30]. Utilising this approach, we have screened over 8,000 low molecular weight compounds from a drug discovery collection for their antiviral activity against NiV infection. This method facilitated the rapid identification of twenty-eight potential NiV antivirals including three commercially available compounds with IC_50 _values in the nanomolar range. To further validate surrogate assay approaches, we have also confirmed efficacy using a recently described NiV-G-VSV-pseudotype assay which mimics multicycle replication [32].

Gentian violet (a.k.a. crystal violet) was introduced as an antiseptic by Sterling in 1890 and is used at a concentration of 1-2% in aqueous solutions [40]. Gentian violet is a cationic triphenylmethane dye which has been used in medicine for its antibacterial, antifungal, and antiparasitic activities (Reviewed in [41]) and has also been used as a mycostatic agent in poultry feed [42]. Gentian violet inhibits DNA replication in a number of bacteria [43] and several hypotheses have been provided to explain the selective toxicity of gentian violet in bacteria and trypanosomes (reviewed in [41]) including alteration of the redox potential by the dye, inhibition of protein synthesis, disruption of Ca^2+ ^homeostasis and a photodynamic action of gentian violet has been described in both bacteria and Trypanosoma cruzi. Gentian violet has been shown to depress protein synthesis in fibroblasts *in vitro *[44] and Hoffmann and co-workers [45] found that gentian violet is a potent inhibitor of amino acid transport and that this inhibition is apparently responsible for its inhibitory effect on T. cruzi protein synthesis.

Recently, Nagayama [46] examined the antiviral activity of gentian violet and gentian violet-dyed cloth against the influenza A (H1N1) virus. When 10^6 ^TCID_50 _virus was exposed to 0.0063% (~160 μM) gentian violet, the residual viable count decreased to below three logs within 30 min and below five logs at 60 min. This indicates that the interaction of gentian violet with the influenza virus is very rapid and gentian violet completely destroys the infectivity of the influenza virus within 60 min. Electron microscopy of gentian violet treated viral envelopes confirmed destruction by gentian violet. While we did not observe clear inhibition of an H1N1 virus in the current study, cellular toxicity prevented effective testing of concentrations greater than 100 μM. The interaction of cationic dyes with cellular membranes has been established for many years [47-50] and for this reason they have been applied in the study of membrane function in mitochondria or intact plasma membranes. Antiviral efficacy may be due to cationic dyes binding directly to the membranes causing perturbation of the membrane structure [51] as lipid bilayers are solvents for apolar and amphipathic compounds such as gentian violet [52,53].

Previous studies suggest the potentiation of the antiviral effects of gentian violet (and the related brilliant green) when applied following NiV infection could be attributable to either a direct interaction with viral and/or cellular membranes or via a general decrease in protein synthesis. Gentian violet did induce an immediate increase in intracellular calcium concentrations and a large decrease in sodium levels suggesting the integrity of cellular membranes may have been compromised (data not shown) but did not induce significant changes in either IL-8 or TNF-α expression. Preincubation of cells with gentian violet prior to virus infection does reduce the expression of viral protein (and by inference, a proportional decrease in viral replication) but does not appear to differentially effect viral replication when preincubated with virus, or when applied during or immediately after virus infection. It is likely that any effect due to direct interaction with cellular membranes should be comparable both during and post-infection with the caveat that post-infection provides a greater time span for this interaction to occur.

Brilliant green (a.k.a. malachite green) has also been used as an antiseptic, similar to gentian violet. The value of certain triphenylmethane dyes such as brilliant green and gentian violet as selective agents for isolation of typhoid bacteria was first reported by Drigalski and Conradi (Reviewed in [54]). These dyes have since been used extensively as aids in the isolation of bacteria of the typhoid and paratyphoid groups (Salmonella). Brilliant green inhibits the growth of bacteria at lower concentrations than most other dyes and is by far the most widely used dye in selective media (reviewed in [54]). Bakker and colleagues [55] demonstrated inhibitory activity against streptococcus, proteus and staphylococcus spp. in addition to candida albicans. Brilliant green has been used widely as an anti-fungal agent in fish hatcheries [56-58] (Reviewed in [58]) but in recent years the use of brilliant green in aquaculture has been banned in several countries due to accumulating evidence of genotoxic and carcinogenic effects [59-62]. However, a recent study by Bahna and co-workers [63] evaluated a combination of low concentrations of both brilliant green and chlorhexidine *in vitro *as an alternative to alcohol based mouthwashes for preventing oral cavity infections in immunocompromised and cancer patients suggesting opportunities may still exist for brilliant green based therapeutics.

The enhanced efficacy of brilliant green when preincubated with cells and/or virus would suggest potential intercalation into, and disruption of both cellular and viral membranes as potential modes of action. We observed a rapid and sustained increase in intracellular calcium and sodium concentrations with an associated decrease in pH (data not shown) also supporting this possibility. Additionally, brilliant green induced a 15-20 fold increase in TNF-α and IL-8 expression, respectively, suggesting the stimulation of a considerable inflammatory response. The similar efficacy seen with a NiV-G pseudotyped virus, the parent VSV, and HPIV3 indicates brilliant green's antiviral activity is likely not specific to henipavirus entry although we did not observe antiviral efficacy against an influenza virus.

Gliotoxin activity against various bacteria and fungi has been known for some time (Reviewed in [64]) and the first report of antiviral activity was made by Rightsel and co-workers [65] describing activity against poliovirus type 2, herpes simplex virus and against influenza A virus, the latter confirmed in the current study. Further studies reported the antiviral activity of gliotoxin against numerous viruses including poliovirus types 1, 2 and 3, rhinovirus strain HGP, ECHO virus types 12 and 28, measles virus, coxsackie virus, Sendai virus, influenza virus and Newcastle disease virus [64,66]. Subsequent studies identified the antiviral action against poliovirus as being due to the inhibition of viral RNA replication [67], specifically via actions on the poliovirus polymerase 3D^pol ^[68]. The observation in the current study that gliotoxin exerts its effects independently of addition prior to or immediately following virus infection, suggests an action subsequent to viral binding and entry, such as replication, confirmed by our pseudotype data. Consistent with the reported immunosuppressive actions of gliotoxin, we observed a decrease in TNF-α expression in Vero cells following gliotoxin treatment. Pre-incubation of compound with cells prior to virus infection may enable efficacious levels of gliotoxin to enter and remain inside the cell, reducing any potential differences expected between pre-infection and post-infection treatment. Efficacy seen with pre-treatment of virus prior to infection of cell monolayers may indicate a direct interaction with one or more viral proteins such as the viral polymerase. Traditionally, the usefulness of gliotoxin and related fungal metabolites has been limited by their toxicity. However, studies highlighting the potential of gliotoxin as an anticancer agent [69,70] may provide important research into the development and evaluation of less toxic analogues of gliotoxin.

## Conclusion

In the current study we have screened over 8,000 small molecules for antiviral activity and demonstrated potent antiviral activity of three commercially available compounds against NiV and HeV, recently emerged BSL4 pathogens for which no vaccine or therapeutic indications exist. Despite the known toxicity associated with these compounds, gentian violet has been, and still is, used extensively for a range of topical applications. In our quest to discover novel antiviral agents that may be amenable to oral or parenteral administration in the event of acute viral exposure, the three compounds described here may prove excessively toxic for systemic use. However, their use in topical applications for inactivation of viruses in field situations or in hospital settings may warrant further investigation. Additionally, gliotoxin, given its identified actions as a viral polymerase inhibitor, may also provide an important parent molecule with which to develop second generation, non-toxic polymerase inhibitors. This proof-of-concept study demonstrates the utility of a live virus HTS approach for identifying potential antiviral compounds. While all novel drug development is a costly and time-consuming process, eliminating additional live virus confirmation steps required to validate leads identified by surrogate assay screening programs will clearly reduce both the development time and the number of false positives generated. However, the considerable cost and biosecurity advantages of surrogate screening approaches will ensure they have a place in antiviral discovery efforts. As evidence of the comparable results obtained through pseudotyped virus screening, our collaborative group recently identified chloroquine as an effective inhibitor of HeV and NiV in vitro [32] in a primary pseudotype screen, followed by live virus confirmation. In the current study, to further validate this approach, we have confirmed compound efficacy against live virus infection with that observed using a novel VSV pseudotype assay mimicking multicycle replication. While the three compounds reported in this study may only be useful for topical administration, or as disinfectants, this screening approach has also identified a number of promising novel candidate antivirals [38] to be evaluated as potential therapeutics for these currently untreatable, lethal pathogens.

## Materials and methods

### Virus and cells

African Green Monkey Kidney (Vero) cells were grown in Minimal Essential Medium containing Earle's salts (EMEM), antibiotics (100 U Penicillin, 100 μg/ml Streptomycin and 500 μg/ml Fungizone) and 10% foetal calf serum (FCS), designated EMEM-10. 293T (human kidney epithelial) cells were grown in Dulbecco's modified Eagle's medium (DMEM; Mediatech-Cellgro) supplemented with 10% fetal bovine serum and antibiotics at 37°C in 5% CO_2_. All transfections and pseudotype infection experiments were performed in OptiMEM (Invitrogen) supplemented with antibiotics. NiV was isolated in Vero cells from the brain of a human fatally infected in the 1998-99 Malaysian outbreak and was passaged three times in Vero cells then double plaque purified and passaged a further three times in Vero cells as previously described [71]. HeV was isolated in Vero cells from the lung of a horse infected in the Brisbane outbreak in October 1994 and was passaged five times in Vero cells followed by triple plaque purification and a further five passages in Vero cells as previously described [72]. HeV and NiV stock titer were adjusted to 1 × 10^6 ^TCID_50_/ml.

For titrations, serial ten-fold dilutions of samples were made in EMEM and 25 μl transferred to five wells of a 96-well microtitre plate. Vero E6 cells in EMEM containing 10% foetal calf serum were added (2 × 10^4 ^cells/well). Plates were incubated at 37°C for 5-7 days and wells displaying cytopathic effect were scored as infected. Virus titre was calculated using the Reed-Meunch method [73] and the limit of detection was 126 TCID_50_/ml virus. All work with live virus was carried out under Biosafety Level 4 (BSL-4) conditions.

Titers of human parainfluenza virus type 3 (HPIV_3_) virus stocks was assessed by plaque assay performed as described previously [74] while the titer of influenza A/swine/Rachaburi/2000 (H1N1) was determined by end point titration in Vero cells.

### Nipah virus infection of cells and library screening

Vero cells were seeded at a density of (2 × 10^4^) into individual wells of 96-well microtitre plates and incubated at 37°C overnight in 100 μl EMEM-10. Prior to NiV inoculation, media was discarded and 100 μl of 20 μM of different test compounds were added to each well in triplicate. Under BSL4 conditions, 1,000 TCID_50 _of virus in EMEM-10 were added to each well of Vero cells in volumes of 100 μl diluting the final test compound concentrations to 10 μM. After an overnight incubation at 37°C, the culture medium was then discarded, plates were immersed in ice-cold absolute methanol, enclosed in heat sealed plastic bags and the bags surface sterilized with Lysol during removal from the BSL4 laboratory. Methanol-fixed plates were air dried at room temperature for a minimum of 30 min prior to immunolabeling.

### HTS Immunolabeling assay

Assays were performed as previously described [30]. Briefly, plates were washed 3 times with Phosphate Buffered Saline containing 0.05% Tween-20 (PBS-T). Plates were then protein blocked with 100 μl of 2% skim milk in PBS-T and incubated at 37°C for 30 min. After protein blocking, plates were washed 3 times with PBS-T, followed by incubation with 100 μl anti-NiV antibody (rabbit polyclonal anti-N [75] complements of Brian Shiell) diluted 1:1,000 in PBS-T containing 2% skim milk for 30 min at 37°C and then washed 3 times with PBS-T. Plates were incubated with 1% H_2_O_2 _(Sigma) for 15 min at room temperature then washed with PBS-T 3 times.

100 μl of anti-rabbit conjugated HRP (Sigma) diluted 1:2,000 in PBS-T containing 2% skim milk, were added to each well and plates incubated at 37°C for 30 min then washed 3 times with PBS-T. For detection, 100 μl of Chemiluminescent Peroxidase Substrate-3 (CPS-3, Sigma) diluted 1:10 in Chemiluminescent assay buffer (20 mM Tris-HCl, 1 mM MgCl_2_, pH = 9.6) were added to all wells. Plates were incubated at room temperature for approximately 15 min, and then read using a Luminoskan Ascent luminometer (Thermo Fisher Scientific, Waltham, USA) using 100 mSec integration per well.

### Antiviral lead identification and toxicity testing

Test compounds were initially screened in triplicate (10 μM) and those exhibiting 90% or greater reduction in NiV infection (compared to untreated control wells) were designated as antiviral leads. Following the initial screening and identification of leads, the selected compounds were further characterised to determine their IC_50 _against both NiV and HeV *in vitro*, in addition to their 50% cytotoxicity (CC_50_) concentrations. For antiviral assays, half-log dilutions (20 μM-63 nM) of each lead compound were assayed against NiV and HeV as described above. Measurements were collated and non-linear regression analysis performed using GraphPad Prism software (GraphPad Software, San Diego, CA, USA) to determine the IC_50_.

Compound cytotoxicity was determined using both the CellTiter-Glo^® ^cytotoxicity kit (Promega, Madison, USA) in Vero cells and alamarBlue^® ^dye (Invitrogen, Carlsbad, CA, USA) in 293T cells, as per the manufacturer's instructions. Vero cell cytotoxicity was determined in monolayers (40,000 cells) in 96 well plates incubated with half-log dilutions of 200 μl each compound in EMEM-10 (20 μM-63 nM, n = 3) overnight at 37°C. Media was removed and 100 μl of CellTiter-Glo^® ^Reagent, diluted 1:5 with chemiluminescent assay buffer, was added to each well, mixed well to lyse cells, equilibrated to room temperature for 10 min, and then read using a luminometer as described above. 293T cell cytotoxicity assays were performed with half-log dilutions of 80 μl each compound in OptiMEM (4 μM-1 nM, n = 4) incubated overnight at 37°C with a suspension of 10,000 cells in 384 well plates containing a 1:8 dilution of alamarBlue^® ^dye. Fluorescence was then read using a Perkin-Elmer EnVision multi-function plate reader with an excitation filter of 535 nm and a 590 nm emission filter. Non-linear regression analysis was performed using GraphPad Prism software to determine the CC_50_. To evaluate the margin of safety that exists between the dose needed for antiviral effects and the dose that produces unwanted and possibly dangerous side effects (cytotoxicity), the therapeutic index for each lead compound was then calculated from the efficacy and cytotoxicity data (CC_50_/IC_50_).

### Multicycle replication pseudotyped virus infection assays

The VSV-ΔG-RFP is a recombinant VSV derived from the cDNA of VSV Indiana, in which the G gene is replaced with the RFP gene. We obtained VSV-ΔG-RFP complemented with VSV G from Michael Whitt (University of Tennessee Health Science Center). Pseudotypes with NiV F and G were generated as previously described for HeV [34,76]. Briefly, 293T cells were transfected with either VSV-G (gift from M. Whitt), HeV-G/F or NiV-G/F. 24 hrs post-transfection, the dishes were washed and infected (MOI of 1) with VSV-ΔG-RFP complemented with VSV G. Supernatant fluid containing pseudotyped virus (HeV-G/F, NiV-G/F or VSV-G) was collected 18 hrs post-infection and stored at -80°C. For infection assays, HeV-G/F, NiV-G/F or VSV-G pseudotypes were used to infect 293T cells transfected with the corresponding G and F plasmids in addition to a VENUS-YFP construct in the absence of serum as previously described [31]. Briefly, compounds were added in a 5 μl volume into 384-well polystyrene black/clear bottom plates in serial 2-fold dilutions. A 70 μl volume of 10^4 ^293T cells that had been transfected with plasmids encoding NiV, HeV or VSV G and F, and also with Venus-YFP was then dispensed using a Multidrop Combi dispenser (Thermo Labsystems), followed by addition of 5 μl of pseudotyped virus. Plates were incubated at 37°C for 48 hr and then read for two channel fluorescence intensity in a Perkin-Elmer EnVision multi-function plate reader. For detecting RFP expression levels, the wells were read from the top with a 535 nm (40 nm bandpass) excitation filter and a 579 nm (25 nm bandpass) emission filter. For detection of YFP expression, the wells were read from the bottom with a 510 nm (10 nm bandpass) excitation filter and 535 nm (25 nm bandpass) emission filter. Additionally, to ensure the assays were not contaminated with bacteria, an additional read of absorbance at 590 nm was performed. Measurements were collated and non-linear regression analysis performed using GraphPad Prism software (GraphPad) to determine the IC_50 _(RFP) or the CC_50 _(YFP).

### Human parainfluenza virus type 3 (HPIV3) assays

A 5 μl volume of compounds were added into 384-well polystyrene black/clear bottom plates in serial 2-fold dilutions. A 70 μl volume of 10^4 ^293T cells were dispensed as above, followed by the addition of 5 μl of HPIV3 (m.o.i. = 0.8). Plates were incubated for 24 hr followed by immunodetection of viral antigen using a cell monolayer ELISA based assay. Briefly, 10 μl of 37% formalin was added to wells for 10 min (final concentration ~4%). Cells were then washed 3× with PBS, blocked with 80 μl 0.5% BSA and 0.1% sodium azide in PBS for 30 min, washed again and incubated for 60 min with 20 μl anti-HPIV3 serum (Matteo Porotto, diluted 1:200 in PBS). Cells were washed again, incubated with 20 μl protein-G-HRP conjugate (Pierce, Rockford, IL.) for 30 min, then background peroxidase activity was quenched with two 20 min incubations with chemiluminescent substrate (CPS-3, Sigma diluted 1:30 in PBS) followed by visualisation with the same substrate diluted 1:5 in PBS. Luminescence was read using the same multi-function plate reader as the previous assay. Measurements were collated and non-linear regression analysis performed using GraphPad Prism software (GraphPad) to determine the IC_50_.

### Influenza assays

Compounds were serially diluted in EMEM-10 and 25 μl was added to white 96 well plates containing 4 × 10^4 ^Vero cells followed by 25 μl of Influenza A/swine/Rachaburi/2000 (H1N1). Plates were incubated for 24 hrs followed by detection of neuraminidase (NA) activity as a surrogate for viral infection using the NA-Star^® ^luminescent detection kit (Applied Biosystems). Briefly, 10 μl of media from each well was added to 40 μl NA-Star^® ^assay buffer, incubated with 10 μl of NA-Star^® ^substrate for 30 min at room temperature, followed by addition of 60 μl of Accelerator solution and luminescence was read immediately. To determine the direct effect of compounds on NA activity, 25 μl of compound and 25 μl of virus were incubated for 30 min at 37°C, followed by addition of 10 μl of NA-Star^® ^substrate for 30 min at room temperature, addition of 60 μl of Accelerator solution and luminescence read as above.

### Viral RNA isolation and Taqman PCR

After overnight virus infection viral media was removed from cells and 150 μl cell lysis buffer (RLT, Qiagen, containing 0.1% β-mercaptoethanol) was added directly to wells in 96 well plates. The cell lysate was aspirated into PCR tubes and removed from the BSL4 laboratory. RNA was extracted using the Qiagen RNeasy Mini kit as per the manufacturer's instructions. RNA was eluted in a final volume of 50 μl RNase free water. Samples were stored at -20°C prior to Taqman PCR analyses.

The specific NiV Taqman primers, probes and reaction conditions were used as previously reported [17,23]. All Taqman PCR oligonucleotide primer and probe sequences used in this study are available on request.

Assays were performed in triplicate using a one-step protocol consisting of an initial reverse transcription reaction followed immediately by cDNA amplification. All Taqman reagents were purchased from Applied Biosystems except the primers, which were obtained from Geneworks. RNA (2 μl) was added to 23 μl of PCR mix in each well of a MicroAmp optical reaction plate containing 12.5 μl of Taqman One-Step PCR Mastermix, 0.625 μl of 40× Multiscribe/RNase inhibitor, 5.75 μl of distilled water, 1.25 μl each of 18 μM NiV or HeV forward and reverse primers, 1.25 μl of 5 μM HeV or NiV FAM-labeled probe, 0.125 μl each of 10 μM 18SrRNAF and 18SrRNAR, and 0.125 μl of 40 μM 18SrRNA-VIC-labeled probe. The samples were amplified in a GeneAmp 7500 sequence detection system (Applied Biosystems) using the following program: 48°C for 30 min, 1 cycle; 95°C for 10 min, 1 cycle; and 95°C for 15 s and 60°C for 60 s, 45 cycles. To correct for sample variation, C_T _values for viral genome in samples were normalized against 18S rRNA expression and expressed as normalised C_T _values.

### Cytokine analysis

Briefly, vero cell monolayers in 48 well microplates (in triplicate) were treated with either brilliant green, gentian violet or gliotoxin (1 μM) or DMSO control (10 μM). Following overnight incubation RNA was extracted with the Qiagen RNeasy kit according to the manufacturers instructions in a final volume of 40 μl. Eight μl of RNA from each extraction was then digested with 1 unit of DNAse (Invitrogen) for 15 minutes at room temperature and subsequently inactivated for 10 minutes at 65°C according to manufacturer's instructions. The RNA was then reverse transcribed using the Superscript II (Invitrogen) kit. The cDNA samples were diluted 1:5 and were assayed in triplicate for each gene of interest (TNF-α and IL-8) with a SYBR green real time PCR kit (Sigma) using a total reaction volume of 25 μl An ABI Prism 7900HT cycler was used with the following cycling conditions: 95°C for 10 min, 1 cycle, 95°C for 15 s and 60°C for 60 s, 40 cycles. GAPDH levels were measured in duplicate for each cDNA sample to normalize C_T _values for subsequent comparison and calculation of fold change in gene expression over untreated cells. Primers for TNF-α and IL-8 were obtained from SABiosciences.

## List of Abbreviations

BG: brilliant green; BSL: biosafety level; CC_50_: concentration resulting in 50% cytotoxicity; DAPI: 4',6-diamidino-2-phenylindole; GV: gentian violet; glio: gliotoxin; HeV: Hendra virus; HRP: horseradish peroxidase; HRP: horseradish peroxidase; HTS: high throughput screening; IC_50_: concentration resulting in 50% inhibition; moi: multiplicity of infection; NiV: Nipah virus; PBS-T: phosphate buffered saline containing tween; TCID_50_: 50% tissue culture infectious dose; VSV: vesicular stomatitis virus.

## Competing interests

The authors declare that they have no competing interests.

## Authors' contributions

MA performed much of the live Nipah and Hendra virus antiviral assays and drafted the manuscript, MLS performed many of the pseudotype assays, MKL performed all the cytokine analysis, CLR performed the influenza antiviral assays, MP assisted with pseudotype assays and manuscript preparation, AGM and SS provided compound library support and contributed to the study design, AM provided laboratory and funding support for the pseudotype assays and contributed to manuscript preparation, BAM provided the study design, assisted with live virus and pseudotyped assays, provided funding support and coordinated manuscript preparation. All authors have read and approved the final version of this manuscript.
